# Fatigue in chronic inflammation - a link to pain pathways

**DOI:** 10.1186/s13075-015-0784-1

**Published:** 2015-10-05

**Authors:** Karine Louati, Francis Berenbaum

**Affiliations:** Department of Rheumatology, Assistance Publique-Hôpitaux de Paris (AP-HP), Saint-Antoine Hospital, F-75012 Paris, France; Inflammation-Immunopathology-Biotherapy Department (DHU i2B), Pierre & Marie Curie University Paris 06 - INSERM UMR_S 938, Paris, France

## Abstract

Fatigue is a frequent symptom in several inflammatory diseases, particularly in rheumatic diseases. Elements of disease activity and cognitive and behavior aspects have been reported as causes of fatigue in patients with rheumatoid arthritis. Fatigue could be associated with activity of inflammatory rheumatism. Indeed, biologic agents targeting inflammatory cytokines are effective in fatigue. Fatigue is also associated with pain and depressive symptoms. Different pathways could be involved in fatigue and interact: the immune system with increased levels of pro-inflammatory cytokines (interleukin-1 and −6 and tumor necrosis factor alpha), dysregulation of the hypothalamic-pituitary-adrenal axis and neurological phenomena involving the central and autonomic nervous systems. A pro-inflammatory process could be involved in pain and behavioral symptoms. Inflammation could be a common link between fatigue, pain, and depression.

## Introduction

Fatigue is usually defined as a state of exhaustion and decreased strength accompanied by a feeling of weariness, sleepiness, and irritability, with a cognitive component [1]. A physiological fatigue state, occurring after strong physical effort, sends a signal to the body to bring it to rest to rescue the exhausted tissues (that is, the muscles). Unlike normal fatigue, pathological fatigue does not improve with rest. This kind of fatigue is seen in most acute and chronic inflammatory diseases, including arthritis.

This review discusses the place of fatigue in various inflammatory diseases but also the possible link with inflammation, pain and depression. We explain this relationship in terms of physiopathologic mechanisms and discuss how inflammation could have a role in the three other domains - fatigue, stress or depression, and pain. We searched for articles in MEDLINE via PubMed with the key words ‘inflammation’ , ‘fatigue’ , ‘pain’ , ‘depression’ , ‘rheumatologic diseases’ , ‘chronic fatigue syndrome’ and ‘treatment’. The search was completed by a hand search of references of the most relevant studies or published reviews.

## Multidimensional and multicausal aspects of fatigue

Fatigue is a multidimensional concept and has various causes. In rheumatic diseases, the association between fatigue and pain has been well established [2–4]. High fatigue is most often associated with high pain, and fatigue and pain seem to be synchronous [2, 5]. The link between fatigue and disease activity is less clear. First, fatigue is clearly a symptom included in rheumatic diseases: in rheumatoid arthritis (RA), it is an important outcome to evaluate according to OMERACT [6], and it has been associated with the Disease Activity Score in 28 joints (DAS28) and the Clinical Disease Activity Index [7]. In spondyloarthritis (SpA), fatigue is part of the Bath Ankylosing Spondylitis Disease Activity Index (BASDAI) and appears more strongly related to the disease process than patient-related variables [8]. Furthermore, in anti-neutrophil cytoplasmic antibody-associated vasculitis, fatigue was associated with increased levels of C-reactive protein (CRP) [9]. However, a systematic review of fatigue in RA found no link between it and some characteristics of inflammatory activity, such as erythrocyte sedimentation rate or DAS28 [2]. Of note, Lee et al. [10] described a subgroup of RA patients with well-controlled disease but high persistent levels of fatigue (34 %).

**Fig. 1:**
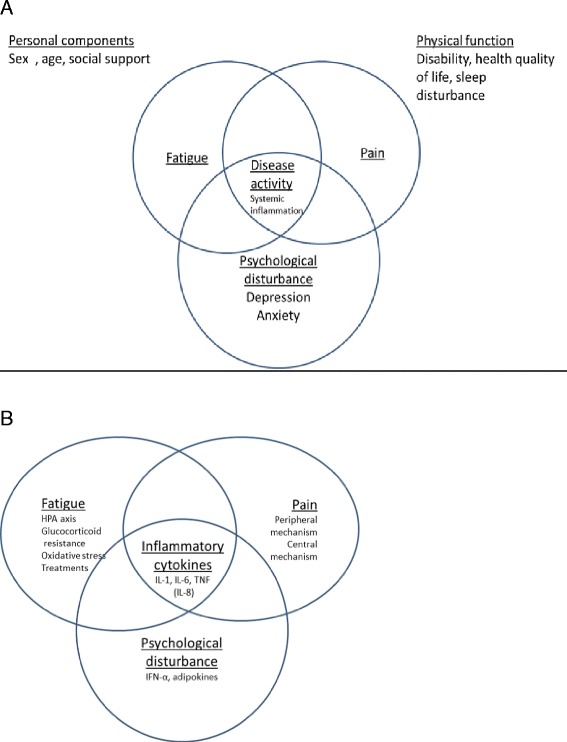
Model of interactions between fatigue, pain and psychological disturbance. (**a**) Conceptual model of clinical interactions between fatigue, pain and psychological disturbance in inflammatory arthritis. (**b**) Conceptual model of physiological interactions between fatigue, pain and psychological disturbance in inflammatory rheumatism. The potential mechanisms of action in each domain are listed. The increase of inflammatory cytokine levels could be involved in fatigue, pain and mood disorders. HPA, hypothalamic-pituitary-adrenal; IFN, interferon; IL, interleukin; TNF, tumor necrosis factor

These discrepancies could be explained by the variable definitions of fatigue or its multidimensional aspect. Indeed, several aspects can affect fatigue: illness-related characteristics (pain, inflammation, disease activity and joint damage), physical functioning (disability, health-related quality of life, sleep quality), cognitive and emotional impairment (anxiety and depression) and personal components (gender, age, social support, work and environment) (Fig. 1a) [2, 11–14]. The multidimensional nature of fatigue was well described by Hewlett et al. [13] in their conceptual model of the interaction between fatigue and three components - disease process, cognitive and behavior aspects, and personal life issues - with a bidirectional path suggesting interrelationships among these components. In this model, pain was included in the disease process and could cause fatigue but could also interact with other factors such as the inflammatory process (responsible for pain, joint damage and disability), anemia and sleep disturbance [2, 9, 13–15]. Conversely, in RA, fatigue seemed associated more with the global assessment of RA or pain than inflammatory components such as erythrocyte sedimentation rate or swollen joint count, perhaps because of the effect of sleep disturbance or reduced physical activity on fatigue [3, 4, 16, 17]. Among the other components of the Hewlett et al. model, cognitive and behavioral factors interacted with thoughts, feelings, behaviors, and symptoms [13]. More recently, Rongen-van Dartel et al. [18] showed a high level of daily physical activity was associated with reduced fatigue even after adjustment for pain and other confounding factors. In SpA, high fatigue was associated with the disease process, such as high disease activity (BASDAI), but also personal components, such as poor quality of life, and other disease activity (bowel symptoms) [19, 20]. Therefore, fatigue is a subjective symptom that interacts with the multiple aspects involved in inflammatory diseases.

## Fatigue and inflammation

Fatigue is common among individuals living with a chronic illness, particularly a disease with an overriding inflammatory process: rheumatologic diseases such as RA or SpA, cancers, inflammatory bowel diseases, connective tissue diseases such as systemic sclerosis, systemic autoimmune diseases, autoimmune type 1 diabetes and infections [14, 21, 22]. In a descriptive study, fatigue was more severe in patients with inflammatory bowel disease than in controls whatever their age [23].

In patients with cancer (acute myelogenous leukemia and myelodysplastic syndrome), fatigue severity was correlated with serum levels of the inflammatory cytokines interleukin (IL)-6, tumor necrosis factor (TNF)-α and the IL-1 receptor antagonist (IL-1RA). In lung cancer patients, IL-8 was a relevant genetic factor of pain and fatigue [24, 25]. Moreover, the expression of IL-6 and nuclear factor-kappa B (NFκB) was increased in oncology patients with sleep disturbance [26]. In these patients, inflammation-induced fatigue could be explained by cancer treatments (radiation, chemotherapy) but also by secretion of inflammatory cytokines by the tumor [27]. Even after chemotherapy or radiation treatments, fatigue could persist for up to 10 years, when levels of a number of pro-inflammatory plasma markers, including IL-6, IL-1RA, CRP and soluble TNF receptor type II, were high [27, 28]. A cumulative effect of levels of cytokines corresponded to the number of days with fatigue in both breast and prostate cancer patients [28]. As well, circulating T lymphocytes were increased in number, with no alteration in immune cells [28]. A recent review described fatigue occurring with inflammation before, during and after treatment with several cancers. Fatigue was well correlated with high levels of inflammatory peripheral cytokines (IL-6, IL-1 and TNF), which could signal the central nervous system (CNS) and generate fatigue or other behavioral symptoms [29].

For some authors, the link between inflammation and fatigue is less clear. A subgroup of patients with high fatigue and high depression harbored a minor allele for the anti-inflammatory IL-4 [30]. Moreover, fatigue was associated with some immunotherapies for HIV/AIDS, such as interferon (IFN)-α and IL-2; about half of 317 patients living with HIV/AIDS reported high levels of fatigue. In this study, plasma TNF levels were lower with antiretroviral therapy, CD4+ T-cell counts of at least 200 cells/mm^3^ and undetectable viral loads; however, on multivariable analyses, plasma levels of none of the cytokines evaluated was significantly associated with fatigue [10].

However, the replication of studies showing an association between expression of genes or inflammation cytokines and fatigue regardless of chronic illness suggests that inflammation could play a role in the fatigue experience (Fig. 1b).

## Fatigue in chronic rheumatologic diseases

Unusual and chronic fatigue with various etiologies was described for 27 % of patients in a primary care clinic [31]. Fatigue is common in RA, SpA, Sjögren syndrome, systemic lupus erythematosus and vasculitis, although most publications concerned fatigue in RA or SpA [9, 14, 32–35]. In RA and SpA, the frequency of fatigue ranged from 42 % to 80 % depending on the definition and methods of assessment [1, 4, 8, 36–39]. For 75 % of patients with ankylosing arthritis and 50 % of those with RA, fatigue was considered severe [4, 39].

Several methods of evaluation have been used to investigate fatigue in rheumatologic diseases [14]. The simplest and quickest scale is the visual analog scale (VAS; scores from 0 to 100; the higher the score, the greater the fatigue), but this is a unidirectional scale. Multidirectional scales developed to include the different aspects of fatigue are the Medical Outcomes Study Short Form 36 (SF-36) vitality subscale (four questions; scores from 0 to 100; the higher the score, the lower the fatigue), the Functional Assessment of Chronic Illness Therapy Fatigue Scale (domains physical, social/family, emotional and functional; scores from 0 to 52; the higher the score, the lower the fatigue), the Profile of Mood States, the RA-specific Multidimensional Assessment of Fatigue (MAF) scale, the Multidimensional Fatigue Inventory, the Brief Fatigue Inventory, and the Fatigue Severity Scale [40–42]. With the VAS scale, the mean fatigue level in patients with RA was 42.1 [43].

Classical treatments of RA and SpA consist of pharmacological treatments to control inflammation and multidisciplinary interventions such as cognitive behavioral therapy and physical exercises to reduce symptoms and maximize self-management [44]. Among pharmacological treatments known to reduce disease activity in RA, disease-modifying antirheumatic drugs (DMARDs), such as methotrexate and leflunomide, or biologic treatments, such as anti-TNF (infliximab, adalimumab, etanercept, golimumab and certolizumab), anti-IL-6 (tocilizumab), CTLA4 immunoglobulin (abatacept) and anti-CD20 (rituximab), have improved pain and mood disorders as well as fatigue, although the overall effect size of biotherapies on fatigue was small (effect size = 0.45; 95 % confidence interval 0.31 to 0.58) [4, 45, 46]. More recently, new biologic therapies have shown efficacy for fatigue: FACIT-Fatigue and SF-36 vitality scales were improved with secukinumab, an antibody against IL-17, and tofacitinib, an oral Janus kinase inhibitor [47, 48]. The placebo effect on fatigue was seen in our meta-analysis of the effect of biotherapies on fatigue: scores ranged from 1.04 ± 22.6/100 (DEO19 study) to 11.57 ± 21.92/100 (REFLEX study) [46]. Wells et al. [49] found that 69, 84 and 90 % of American College of Rheumatology 20/50/70 responders receiving abatacept, respectively, had a >20 % improvement in fatigue according to the VAS. So even if the level of concordance between fatigue and disease activity was high, 10 % to a third of patients had fatigue whereas disease activity improved. With secikinumab, however, patients reporting increased pain showed worsened fatigue according to both fatigue scores [48]. After 3 months of anti-TNF therapy for RA, fatigue was decreased in patients but was independent of the level of CRP: on multiple regression, only global health and tender joint count explained 34 % of the variance in fatigue [50]. Otherwise, a randomized controlled trial showed significant benefits of an exercise program on fatigue scores, quality of life, pain and sleep quality [51]. After six weekly sessions of behavioral therapy and a consolidation session, RA patients with initial VAS fatigue score ≥6/10 reported better fatigue scores than controls (MAF and VAS scales) and better perceived fatigue severity, coping, disability, depression, helplessness, self-efficacy and sleep [52].

## Link between fatigue and pain

Fatigue and pain are two common symptoms in RA, and the link between fatigue and pain in it has been described [2–4, 53]. In a cross-sectional study, VAS fatigue score was mainly correlated with VAS pain score; pain was most strongly associated with the five variables explaining fatigue [4]. Garip et al. [53] confirmed this association, showing that fatigue intensity in RA patients was strongly correlated with VAS pain score and DAS28, with greater correlation between fatigue and pain scores than between fatigue score and DAS28. Moreover, after treatments (DMARDs or biologic agents), the decrease in VAS fatigue score was correlated with ameliorated pain and improved DAS28 score [4]. For Wolfe et al. [11], pain was a strong independent predictor of fatigue with sleep disturbance, depression, tender joint count and disability by the health assessment questionnaire. Among the variables usually linked to fatigue, pain was a better predictor of fatigue [54].

To examine the bidirectional effect of pain and fatigue, van Dartel et al. [5] conducted a prospective study of patients with established RA who received DMARDs and/or biologic agents; pain and fatigue were measured monthly for 1 year. Pain and fatigue levels fluctuated, and the change in fatigue level was positively associated with change in pain level during the same month. However, change in fatigue level was not related to a change in pain level that occurred 1 month earlier, and change in pain level was not related to a change in fatigue level 1 month earlier [5]. Moreover, in a cohort of patients with fibromyalgia, usually considered a non-inflammatory disease, pain and fatigue scores were correlated (r = 0.45, *P* < 0.001) and, in the prospective analysis at 1 week, daily pain evaluation predicted increased fatigue level reported the next day (more than depression or daily sleep quality) [16].

## Fatigue and pain pathways: role of inflammation

### Role of inflammation in fatigue

The mechanisms of fatigue are complex and have been studied in animal models and humans. Because fatigue could be explained by loss of muscle mass or altered mood, Norden et al. [55] proposed a model to discriminate between these phenomena: some colon tumor-bearing mice demonstrated signs of fatigue (decreased voluntary wheel-running activity) and depressed mood (resignation and anhedonia), with no association with decreased normalized contractile properties of skeletal muscle of the limb. So fatigue seemed linked more to behavior than muscle activity.

Inflammation could play an important role (Table 1). The injection of IL-1 in murine models decreased social exploration and increased hypersomnia and body weight loss, which were all improved by the administration of anti-inflammatory IL-1RA or IL-10 [56, 57]. Moreover, in the model of tumor-induced fatigue in mice seen earlier, fatigue was associated with increased levels of IL-1 and IL-6 in the brain, and treatment with minocycline, an anti-inflammatory agent, improved grip strength without reducing tumor growth or muscle mass [55].

**Table 1:** Possible mechanisms involved in fatigue

Endocrine system	Dysregulation of HPA axis and resistance to glucocorticoids
	Thyroid insufficiency
Central nervous system	Decrease or polymorphism of neurotransmitters
Peripheral nervous system	Alteration of autonomic system
	Stimulation of vagus nerve via microbiota or inflammation
Anemia	Decrease of tissue oxygenation
Inflammation	Increase of levels of pro-inflammatory cytokines (IL-1, IL-6, IL-8 and TNF)
	Proliferation of immune cells
Oxidative stress	Excess formation of free radicals
Treatments	Possible side effects

*HPA* hypothalamic-pituitary-adrenal, *IL* interleukin; *TNF* tumor necrosis factor

The role of inflammation in fatigue has also been shown in patients. Indeed, in those with chronic fatigue syndrome (CFS), fatigue intensity was associated with high circulating IL-8 levels [58]. Moreover, in an observational study of military personnel with insomnia, CRP level was reduced more in the restorative sleep group than in those with persistent insomnia [59]. In RA patients, a meta-analysis of therapeutic studies showed that inhibiting levels of some pro-inflammatory cytokines by biologic agents such as anti-TNF, anti-IL-6, CTLA4 immunoglobulin or anti-CD20 significantly decreased the level of fatigue whatever the therapy [46].

**Fig. 2:**
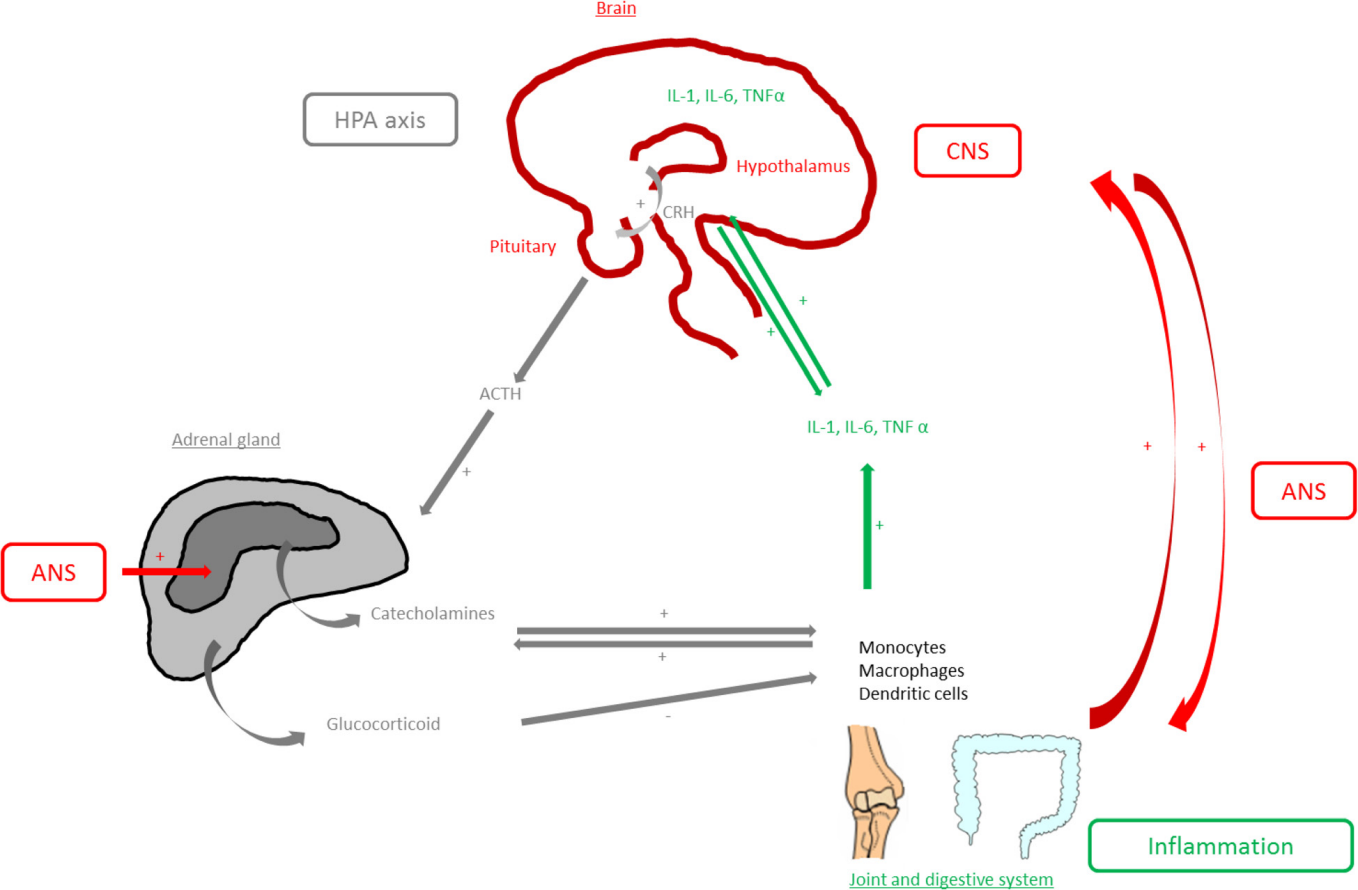
Mechanisms of interaction between peripheral inflammation, the nervous system and the hypothalamic-pituitary-adrenal (HPA) axis involved in the fatigue process. In the HPA axis, the hypothalamus contains neurons that synthesize corticotropin-releasing hormone (CRH), which regulates adrenocorticotropic hormone (ACTH) by the pituitary gland. ACTH stimulates the synthesis of glucocorticoids such as cortisol by the adrenal cortex and catecholamines by the adrenal medulla of the adrenal gland. Cortisol could have a negative feedback mechanism on the brain. Glucocorticoids inhibit many functions of leukocytes and the production of pro-inflammatory cytokines (interleukin (IL)-6 and IL-1) by immune cells. ACTH and CRH have pro-inflammatory properties and IL-1, IL-6 and tumor necrosis factor (TNF)-α activate the HPA axis. The peripheral nervous system can affect inflammation: the sympathetic neurons of the autonomic nervous system (ANS) secrete pro- and anti-inflammatory neuropeptides. These pro-inflammatory cytokines could enter central nervous system (CNS) areas by the permeable blood–brain barrier or facilitate the release of second messengers to induce cytokine activity in the brain. With excess inflammation, the activity of some CNS neurotransmitters could be altered

Otherwise, fatigue could be due to inflammation-induced anemia by decreasing iron levels mediated by IL-6-induced hepcidin and thyroid insufficiency or decreased hypothalamic-pituitary-adrenal (HPA) axis activity and resistance to glucocorticoids (Fig. 2 and Table 1) [27, 60, 61]. In this system, the release of adrenocorticotropic hormone is affected by the sleep cycle, but in some diseases, the circadian cortisol cycle is abnormally flattened [61]. Therefore, neurological phenomena could be involved in fatigue (Table 1). The role of CNS neurotransmitters was mentioned in recent reviews [27, 62, 63]: fatigue was found to be related to polymorphism in catechol-O-methyltransferase (COMT) and low levels of tryptophan, an amino acid involved in the synthesis of serotonin or impaired brain dopamine and norepinephrine transmission [27, 62, 63]. In parallel, the autonomic activity was altered in a model of fatigue induced by a cognitive task, the Kana Pick-out Test (alternating open and closed eyes): VAS fatigue score was associated with decreased parasympathetic and increased sympathetic sinus modulation as evaluated by electrocardiography [64]. Moreover, this model of induced fatigue activated the dorsolateral prefrontal cortex and cingulate cortex as assessed by functional magnetic resonance imaging (MRI) [64, 65].

Systemic inflammation could affect these central mechanisms. Under some circumstances, such as chronic anxiety, posttraumatic stress, and local or general inflammation diseases, the HPA axis was deregulated and the persistent secretion of corticoids induced glucocorticoid resistance [66]. The HPA axis has also been shown to interact with the immune system (Fig. 2) [61]. Moreover, although the brain is considered an immunologically privileged site, systemic infection or inflammation can have a profound effect on the CNS. In an animal model of inflammation, the peripheral administration of lipopolysaccharide increased IFN-stimulated genes in the brain [66, 67]. Peripheral pro-inflammatory cytokines could have a direct action when they enter CNS areas where the blood–brain barrier is permeable and an indirect action when they facilitate the release of second messengers to induce cytokine activity in the brain or when they activate the vagus or other afferent nerves [14]. TNF-α could participate in microglial activation in promoting rolling and adhesion of leukocytes along cerebral endothelial cells, which negatively affects dopaminergic neurotransmission [27, 63, 66–68]. However, anti-TNF agents are unable to penetrate the blood–brain barrier [69]. Inflammatory cytokines would also be responsible for a relative deficit in tetrahydrobiopterin used in the synthesis of the neurotransmitters dopamine, norepinephrine and serotonin [63]. The CNS releases norepinephrine, which is responsible for upregulating IL-1, IL-6 and TNF [66]. However, most studies examined acute inflammation, and the role of neurotransmitters in chronic inflammation is not well established. A bidirectional interaction between the neuroendocrine system and peripheral inflammation could play a role in fatigue.

### Role of inflammation in pain

Pain has been investigated in animal models and humans. In animal models, pain could result from complex interactions between joint inflammation and altered pain processing: a peripheral mechanism (for example, increased innervation of the synovium; increased dorsal root ganglia expression of substrance P, calcitonin gene-related peptide and neuropeptide Y; increased expression of tyrosine kinase receptor A for nerve growth factor and neuronal death) and central mechanism (for example, nociceptive pathway activity, increased sensitivity of spinal neurons via glian, and activation via interleukin (IL-1, IL-6 and TNF), opiod expression in ganglia, central sensitization). Dopamine and serotonin systems are also involved in pain: *COMT* gene expression and polymorphisms of serotonin transporter genes were found associated with pain [62]. Patients with RA showed production of peripheral pain agents, pro-inflammatory cytokines (IL-1, IL-6 and TNF with different actions on responsiveness of Aδ-fibers, C-fibers and the effect of neutralization on mechanical hyperalgesia) and nerve growth factor in synovium or synovial fluid, which sensitized peripheral receptors [70]. TNF-α injected in mouse joints induced persistent sensitization of nociception with noxious stimuli, with a dose-dependent effect, with prevention by injection of an anti-TNF agent [71]. Endogenous opioids, somatostatin, lipid mediators and anti-inflammatory cytokines (IL-4 and IL-10) were also present in synovial tissue, but their roles remain to be determined. Central pain processing was increased in RA patients, with a change in neuronal adaptive response and increased activity of the thalamus, secondary sensory cortex and limbic system, which could be modulated by emotional processing or low mood [70, 72]. Proinflammatory cytokines could have a direct action on pain via sensory neurons or an indirect action via inflammatory mediators such as prostaglandins [70].

### Role of inflammation in altered central nervous system activity

Because fatigue is also often associated with anxiety and depression in inflammatory rheumatism, it may be due in part to a neurological phenomenon. Pro-inflammatory cytokines could be involved: administration of IL-1β, IL-6 or TNF-α in mice increase behavioral symptoms such as social exploration [56, 68]. A review showed that blood levels of some inflammatory cytokines, such as mitogen-stimulated cytokines and adipokines, were higher with depression [68]. A meta-analysis of 24 publications reporting on levels of cytokines in depressed patients found increased levels of TNF-α and IL-6 but not IL-1β, IL-4, IL-2, IL-8, IL-10 or IFN-γ [73]. In some of these studies, however, this association could represent a subset of patients; for example, those with a high degree of depression, who were older and had comorbidities [74]. In older patients of the Rotterdam study, despite no association between blood levels of IL-6 and CRP and depressive symptoms, high levels of these inflammatory proteins predicted depressive symptoms 5 years later [74]. Moreover, in pooling the data for five placebo-controlled trials, Iyengar et al. [75] showed that treatment with nonsteroidal anti-inflammatory drugs (the ibuprofen or naproxen group and the celecoxib group) was associated with decreased depression score and showed a trend to changed classification of depression at 6 weeks. Moreover, antidepressive agents might inhibit the production of pro-inflammatory IL-6 and stimulate anti-inflammatory IL-4, IL-10 and IL-1RA [76]. However, levels of the proinflammatory cytokines could also be altered by stressors or lifestyle factors associated with depression. Indeed, stress caused by major life events such as interpersonal loss or social rejection was associated with levels of pro-inflammatory IL-6 and TNF-α and also CRP, especially in depressed patients [73].

Stress was also associated with high levels of the pro-inflammatory intracellular transcription factors NFκB and inhibitor of kB and modulated genome-wide expression levels [66]. Thus, inflammation and depression seem to be linked, but which one affects the other is difficult to distinguish and probably there is an interaction between both.

## Inflammation: a potential link between fatigue, depression and pain

Although fatigue, stress or depression, and pain have complex and various mechanisms of action, some inflammatory cytokines are found associated with these three domains, so inflammation may be their potential link. The association between these symptoms has been documented in various medical conditions: classical inflammatory diseases such as rheumatic diseases, cancer or infections but also CFS, metabolic disorders or depression, which exhibit low-grade inflammation [77].

Some authors found that in RA, fatigue, mood disorders and pain are clinically associated [2]; IL-1 level was higher in cerebrospinal fluid of patients than controls and the increased level was correlated with fatigue [78]. Biologic treatments are efficacious and can decrease levels of markers of inflammation such as CRP; tocilizumab is especially efficacious because of its action on IL-6 involved in the synthesis of CRP [79]. Thus, decreased systemic inflammation could be one of the mechanisms of action improving fatigue, pain and mood disorders.

Moreover, in a cohort of 1,466 European patients with advanced cancer, increased CRP level was associated with pain and fatigue (rho = 0.154 and 0.197, respectively) [80]. In ovarian cancer patients, diurnal and nocturnal cortisol and plasma IL-6 levels decreased (became normal) during the first year following surgery, and this decrease was associated with decreased fatigue and depression [81].

Another model of the association of fatigue, pain and depression is CFS. Recently, a review showed that chronic inflammation could explain, in part, the sickness behavior [57]. In this pathology, with increased sensitivity to pain and with sickness behavior, inflammatory cytokines could have an effect on nociception. Such cytokines appeared to be critical mediators of hyperalgesia in a lipopolysaccharide-induced animal model [82, 83]. In CFS, the levels of neopterin, a marker of cellular immune system activation, IL-1 and TNF were correlated with fatigue and depression [84]. Brain inflammation could have a role in CFS, in part by activating microglia or astrocytes. Indeed, the density of 11C-(R)-(2-chlorophenyl)-N-methyl-N-(1-methylpropyl)-3-isoquinoline-carboxamide, a marker of neuroinflammation, was increased in some areas of the brain, in particular the cingulate cortex, in patients with CFS compared with healthy patients. Its increased level in the thalamus was correlated but not significantly with pain score and fatigue sensation (*P* = 0.0683) [85].

We have emerging evidence of the role of microbiota in the pathogenesis of autoimmune disease, particularly in rheumatologic disease [86]. Recently, Galland [87] proposed a schema in which the gut microbiome could affect CFS or fibromyalgia: the bacterial components could excessively stimulate the innate immune system and induce systemic and CNS inflammation by producing neurotoxic metabolites or could directly stimulate afferent neurons of the nervous system to send signals to the brain via the vagus nerve. Then, the gut microbiome could affect the HPA axis and be responsible for fatigue and pain in these diseases.

Moreover, it is interesting to highlight brain areas involved in these various domains. With the Kana Pick-out Test, in the model of induced-fatigue previously described, the dorsolateral prefrontal cortex and cingulate cortex were activated as seen on functional MRI [64, 65]. In adults, noxious stimulation increased MRI activity in primary somatosensory cortices, the anterior cingulate cortex, bilateral thalamus, and divisions of the insular cortices [88]. There is a common area for neural activity in experiences of physical pain, induced fatigue, depressive mood and bacterial endotoxin-induced inflammation (with increased IL-6 level): the anterior insula, believed to play a role in consciousness and in emotion or regulation of the body’s homeostasis, and anterior cingulate cortex, known to be involved in autonomic and cognitive functions [64–66]. A common CNS pathway would be a link between fatigue and pain, and among the different mechanisms of fatigue, the action of inflammation on the CNS could be one of these pathways.

## Conclusion

Fatigue and pain are two symptoms frequently present in acute or chronic high-grade inflammatory diseases such as infection, rheumatoid diseases or cancers but also low-grade inflammatory diseases such as CFS. These symptoms are often associated with depression. In all these diseases, several markers of inflammation have been highlighted, and among various and complex mechanisms of action, inflammation could be one of the common links between fatigue and pain among various and complex mechanisms. In rheumatic diseases, decreasing inflammation may improve fatigue and pain. Peripheral inflammation localized at organs is associated with central neurological phenomena. The inflammatory cytokines and cells interact with the CNS: peripheral inflammation can provoke fatigue and pain, and an altered neuroendocrine system could modulate inflammation. Because of multiple aspects of fatigue among patients and multiple mechanisms of action that could be involved, a future perspective would be to identify some phenotypes of fatigue to better target this treatment.

## Note

This article is part of the series ‘*New technologies’*. Other articles in this series can be found at http://arthritis-research.com/series/technology.

## Abbreviations

BASDAI: Bath Ankylosing Spondylitis Disease Activity Index
CFS: Chronic fatigue syndrome
CNS: Central nervous system
COMT: Catechol-O-methyltransferase
CRP: C-reactive protein
DAS28: Disease Activity Score in 28 joints
DMARD: Disease-modifying antirheumatic drug
HPA: Hypothalamic-pituitary-adrenal
IFN: Interferon
IL: interleukin
IL-1RA: IL-1 receptor antagonist
MAF: Multidimensional Assessment of Fatigue
MRI: Magnetic resonance imaging
NFκB: Nuclear factor-kappa B
RA: Rheumatoid arthritis
SF: Short form
SpA: Spondyloarthritis
TNF: Tumor necrosis factor
VAS: Visual analog scale
